# Development and Validation of CXCR4 Nomogram-Based Immune Infiltration/Tumor Inflammation in Primary Glioblastoma

**DOI:** 10.3390/brainsci13081152

**Published:** 2023-08-01

**Authors:** Guannan Jiang, Zong-Qing Zheng, Jie Zhang, Zhichao Tian, Xiang Li, Zhengquan Yu, Zhong Wang, Wanchun You, Gang Chen

**Affiliations:** 1 Department of Neurosurgery & Brain and Nerve Research Laboratory, The First Affiliated Hospital of Soochow University, 188 Shizi Street, Suzhou 215006, Chinawangzhong_8761@163.com (Z.W.);; 2 Institute of Stroke Research, Soochow University, 188 Shizi Street, Suzhou 215006, China

**Keywords:** glioblastoma, C-X-C motif chemokine receptor 4, nomogram, inflammation, immune-cell infiltration

## Abstract

Glioblastoma (GBM) is a highly malignant and aggressive tumor with poor prognosis. Therefore, the discovery of new prognostic molecular markers is of great significance for clinical prognosis. The CXC chemokine receptor (CXCR) members play a key regulatory role in many cancers. In this study, we explore the clinical value and application of the CXCR members in primary glioblastoma. Two GBM datasets from The Cancer Genome Atlas (TCGA) and The China Glioma Genome Atlas (CGGA) databases were used to explore the relationship between differential expression of CXCRs and GBM subtypes as well as immune infiltration. C-X-C motif chemokine receptor 4 (CXCR4) was screened as an independent prognostic factor, and a nomogram and risk prediction model were developed and tested in the CGGA database using the TCGA database. Receiver operating curve (ROC) and decision curve analysis (DCA) found good accuracy and net benefit of the models. The correlation of CXCR4 with immune infiltration and tumor was analyzed using CancerSEA and TIMER. In in vitro experiments, we found that CXCR4 was significantly overexpressed in glioblastoma and was closely related to the inflammatory response of U251/U87 cells. CXCR4 is an excellent independent prognostic factor for glioblastoma and positively correlates with tumor inflammation.

## 1. Introduction

Glioblastoma, which accounts for approximately 15% of all primary brain tumors, is one of the most common and highly lethal central nervous system (CNS) malignancies in adults [1,2,3]. The current treatment option for glioblastoma is surgical resection to relieve neurological symptoms, followed by concurrent radiation and adjuvant chemotherapy [4]. Despite treatments with surgery, radiotherapy, and chemotherapy, the 5-year survival rate of glioblastoma is only 4.7%, and the prognosis is poor [5]. At present, a large number of studies have been done on the prognostic indicators of GBM at home and abroad, but its sensitivity and specificity in clinical application need to be improved. Therefore, there is an acute demand to find reliable novel prognostic biomarkers for GBM to improve the prognosis of GBM.

Over the last decade, many signaling pathways have been investigated as potential targets for GBM therapy [6]. In recent years, it has been reported that some chemokine receptors are involved in tumor growth, proliferation, angiogenesis, and so on. The invasion and development of tumor is an important reason for its occurrence and development [7,8]. A complex regulatory network of CXCR members and their ligands mediates the anti-apoptotic effects on glioma cells and changes in the tumor microenvironment [9]. To date, the CXCR members include CXCR1-7 chemokines [10]. CXCR1 and CXCR2 have comparable affinity to CXCL6 and CXCL8 (IL-8), and CXCR2 mediates tumor angiogenesis [11]. CXCR3 and CCR2 signaling pathways lead to the recruitment of tumor-promoting immune cells such as TAMs, T cells, and myeloid-derived suppressor cells (MDSC) [12]. CXCR4 is highly expressed on tumor cells and can promote tumor cell growth, migration, and invasion [13]. CXCR5 binds to CXCL13 and regulates the growth, proliferation, invasion, and metastatic ability of tumor cells [14]. The CXCL16/CXCR6 axis plays an important role in activating the immune response to glioma cells [15]. CXCR7 mediates the internalization of effective ligands and the degradation of chemokine secretion while regulating the expression of CXCR4 in tumor development [16].

The aim of this study is to investigate the immunological relevance and survival prognosis of CXCR members expression in patients with GBM. First, we applied RNA-seq data from TCGA and CGGA to verify mRNA expression levels of CXCRs in GBM, explore the relationship between CXCRs and GBM subtypes, perform survival analysis, and screen for independent clinical pathologies using Kaplan–Meier and Cox regression analysis. Second, a novel nomogram and prognostic risk model are established, and the biological function of its members is analyzed. This study focuses on exploring the significance of CXCR4 in survival prognosis in GBM and laying the foundation for clinical improvement of GBM outcomes.

## 2. Materials and Methods

### 2.1. Ethics Statement

This trial was supported by the Ethics Committee of the First Affiliated Hospital of Soochow University and approved by clinicians. We obtained informed consent for the use of brain tissue from GBM patients.

### 2.2. Datasets Download

Data from TCGA (obtained from the GlioVis website: http://gliovis.bioinfo.cnio.es/ accessed on 1 January 2023) and CGGA database (www.cgga.org.cn/ accessed on 1 January 2023) (WHO IV primary GBM samples) were used to assess the different CXCRs mRNA expression levels in different subtypes of glioblastoma [17,18]. Sangerbox 3.0 (http://sangerbox.com/login.html/ accessed on 15 February 2023) and the online bioinformatics analysis tools of R (V3.6.3) were used for data analysis.

### 2.3. Functional Enrichment Analysis

Metascape (http://metascape.org/ accessed on 20 February 2023) is an open user-friendly web tool for gene annotation and analysis. It utilizes automated meta-analysis capabilities to process the pathways involved in CXCRs and enrichment analysis of CXCR members and neighboring genes significantly associated with alterations.

### 2.4. Tumor Immunology Analysis

TIMER (https://cistrome.shinyapps.io/timer/ accessed on 25 February 2023) is a web server that provides analysis of the immune system in the filtrate of many cancer types. In this study, we used its compiled module to explore the relationship between CXCRs gene expression and infiltration of six immune cells (B cells, CD4+ T cells, CD8+ T cells, neutrophils, macrophages, and dendritic cells).

### 2.5. Cancer Single-Cell State Atlas

The CancerSEA database (http://biocc.hrbmu.edu.cn/CancerSEA/home.jsp/ accessed on 28 February 2023) includes 41,900 single-cell functional annotations of 25 tumors to study the relationship between the functional phenotype of genes and various tumors. In this study, we used the CancerSEA database to analyze the relationship between CXCRs and glioblastoma.

### 2.6. Model Construction and Evaluation

First, the Kaplan–Meier and log-rank test were used to investigate the relationship between CXCR members and the overall survival (OS) of GBM patients in TCGA and CGGA. CXCR4 with prognostic significance was screened, and its median expression level was used as the cut-off value, combined with other clinicopathological factors (age, gender, IDH mutation status, chemotherapy status, radiotherapy status), and the effect of CXCR4 expression on the prognosis of GBM was analyzed by univariate and multifactorial Cox regression. Then, the nomogram and the predictive risk score models (predictive risk score formula was: Y = 0.421 × Age + 0.393 × CXCR4 − 0.798 × CHEMO − 0.920 × IDH) were constructed to assess the prognosis of GBM patients at 1, 2, and 3 years. The TCGA database was used as the training group (IDH1-WT and IDH1 mutant, *n* = 361) and the CGGA database was used as the external validation group (IDH1-WT and IDH1 mutant, *n* = 175). Finally, the performance of the predictive risk score in GBM was examined using the TCGA database (IDH1-WT, *n* = 338) and the CGGA database (IDH1-WT, *n* = 145) to determine their accuracy. Data visualization was performed using R (V3.6.3) software and an online bioinformatics analysis tool (http://sangerbox.com/login.html/ accessed on 1 March 2023).

### 2.7. Immunohistochemistry Staining (IHC) and Quantification

Tissue paraffin sections were sequentially washed three times with distilled water after passing xylene twice and 100, 90, 80, and 70% ethanol. After antigen repair, endogenous peroxidase activity was blocked with H2O2 (0.3%) for 15 min. It was incubated overnight at 4 °C with CXCR4 antibody (1:100, AF6621, Beyotime, Nantong, China). After washing, the tissue was incubated with the indicated secondary antibody for 30 min. Finally, the sample was re-stained with hematoxylin, dehydrated, and viewed under a microscope. Its quantitative method refers to previous studies [19].

### 2.8. Cell Culture with CXCR4 Knockdown

The U87 and U251 glioma cell lines (purchased from Shanghai Zhongqiao Xinzhou Biological Co., Ltd., Shanghai, China) were cultured in DMEM medium containing 10% FBS at 37 °C. We inoculated U251 and U87 in culture dishes and infected CXCR4 knockout plasmids for 2 d according to the protocol provided by the manufacturer.

### 2.9. Western Blot

Proteins (30 ug) were extracted from whole cell lysates and separated on 10% SDS-PAGE gels as described previously [20]. The protein ribbons were then transferred to the PVDF membrane and closed with a PBS containing 5% BSA for 1 h. After incubation with CXCR4 antibody (1:2000, Abcam, Waltham, MA, USA) overnight at 4 °C, the protein band was incubated for 1 h with the corresponding secondary antibody (CST, 6883, Danvers, MA, USA). Finally, we visualized the protein bands using chemiluminescent reagents.

### 2.10. Enzyme-Linked Immunosorbent Assay (ELISA)

The Human TNF ELISA kit was purchased from Fine Test (EH0302). We conducted experiments according to the manufacturer’s recommendations and expressed the data as an average.

### 2.11. Statistical Analysis

All statistical analysis results were generated through R (V3.6.3) and the Web database, using GraphPad Prism 9.0 software for statistical analysis and visualization. We used a *t*-test to compare the differences between the two groups. *p* < 0.05 was considered statistically significant.

## 3. Result

### 3.1. Transcription Level of CXCRs in Patients with GBM

**Figure 1:**
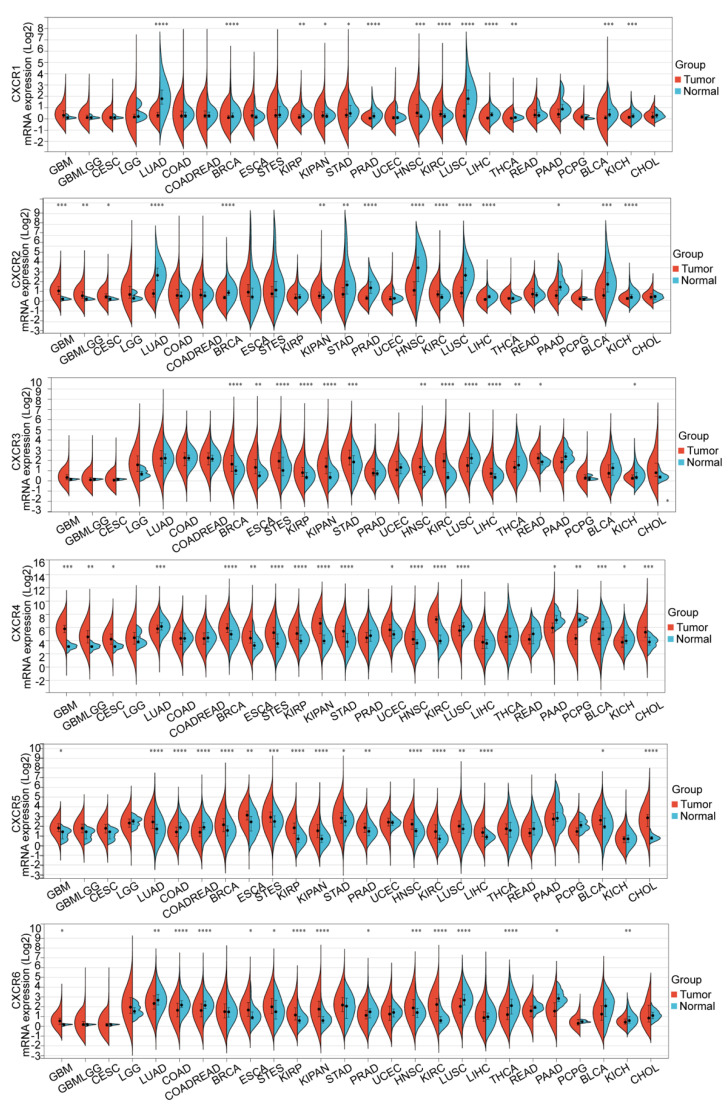
Differential expression of the CXCR members in normal and tumor samples in different cancers. * *p* < 0.05; ** *p* < 0.01; *** *p* < 0.001; **** *p* < 0.0001. The black symbols show the mean values. The full names of the diseases are shown in Table S2 of the Supplementary Materials.

We extracted the expression data of CXCR members (CXCR1/2/3/4/5/6) from TCGA in each sample and calculated the expression differences between normal and tumor samples in each tumor for different subtypes of CXCR members. According to the results (Figure 1), CXCR members were commonly expressed in different pan-cancers; CXCR2 (Tumor: 1.13 ± 1.08; Normal: 0.10 ± 0.07; *p* = 4.8 × 10^4^), CXCR4 (Tumor: 64.05 ± 43.39; Normal: 7.97 ± 2.34; *p* = 2.4 × 10^4^), CXCR5 (Tumor: 2.73 ± 1.49; Normal: 1.35 ± 0.76; *p* = 0.02), and CXCR6 (Tumor: 0.71 ± 0.87; Normal: 0.12 ± 0.05; *p* = 0.02) expressions were significantly upregulated in GBM.

### 3.2. Biological Functional Analysis of CXCR Members

**Figure 2:**
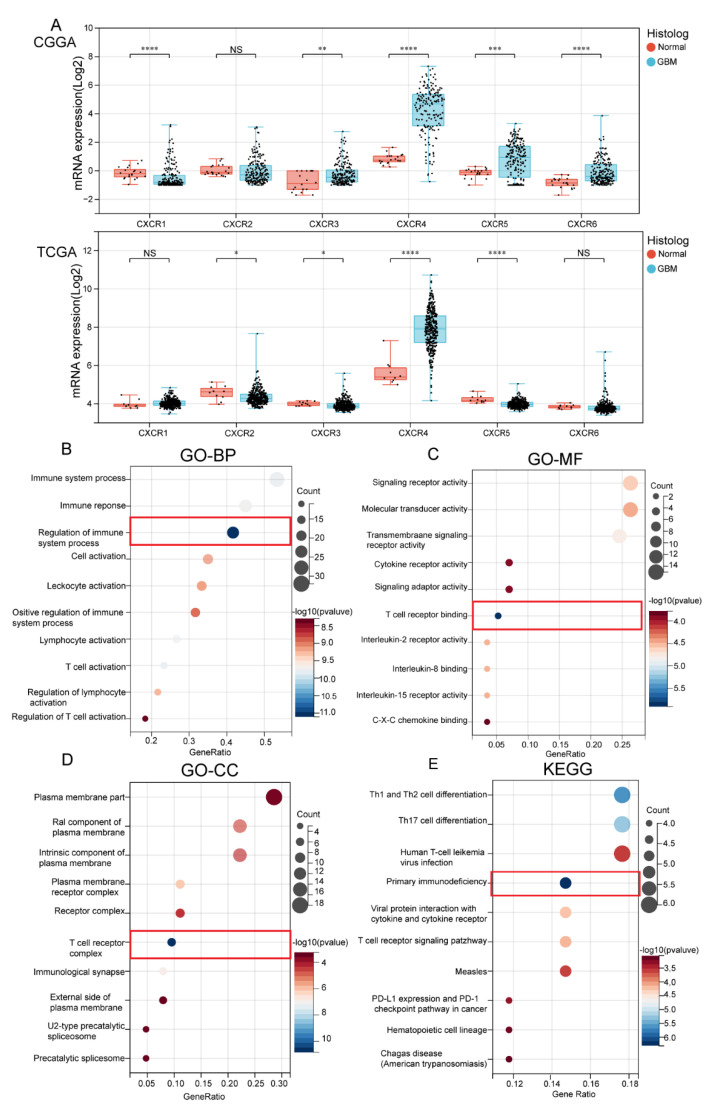
Differential expression and biological functions of the CXCR members in GBM from TCGA and CGGA databases. (**A**), Expression of CXCR members mRNA levels in GBM and normal group groups in TCGA and CGGA databases. The black dots represent the expression of mRNA in the included population. * *p* < 0.05, ** *p* < 0.01, *** *p* < 0.001; **** *p* < 0.0001; ns not significant. (**B**–**E**), Functional enrichment of CXCR members in GBM by GO and KEGG analysis, including biological process (BP), cellular component (CC), molecular function (MF), and KEGG pathway. The red box is the highlighted part.

We integrated and counted data from TCGA and CGGA incorporating WHO IV primary GBM samples (IDH1-WT and IDH1 mutant), as shown in Figure 2A. In the TCGA database, we found that the expression of CXCR2 (Tumor: 4.35 ± 0.37; Normal: 4.56 ± 0.37; *p* = 0.04), CXCR3 (Tumor: 3.92 ± 0.23; Normal: 4.01 ± 0.11; *p* = 0.03), CXCR4 (Tumor: 7.87 ± 1.03; Normal: 5.64 ± 0.67; *p* = 1.0 × 10^6^), and CXCR5 (Tumor: 3.98 ± 0.17; Normal: 4.23 ± 0.18; *p* = 1 × 10^5^) were significant; in CCGA, we found that the expression of CXCR1 (Tumor: −0.48 ± 0.80; Normal: −0.14 ± 0.42; *p* = 9.7 × 10^5^), CXCR3 (Tumor: 0.23 ± 0.76; Normal: 0.78 ± 0.67; *p* = 7.0 × 10^3^), CXCR4 (Tumor: 4.22 ± 1.69; Normal: 0.83 ± 0.34; *p* = 8.6 × 10^11^), CXCR5 (Tumor: 0.74 ± 1.21; Normal: −0.16 ± 0.32; *p* = 7.9 × 10^4^), and CXCR6 (Tumor = −0.10 ± 0.81; Normal: −0.85 ± 0.35; *p* = 5.4 × 10^6^) were significant. Combining the two databases, the mRNA levels of CXCR3,4,5 were found to be significantly higher in GBM tissues than in normal tissues, suggesting that these factors may have a relevant role in GBM tumor development.

To further investigate the specific role of CXCR members in GBM for tumors, we screened the top 20 × 6 genes of each CXCRs in GBM from TCGA database (see Supplementary Materials Table S1). The protein–protein interaction network showed that inflammatory factors (IL-12) and immune cells (e.g., Th1, Th2, and Th17) were most associated with CXCR members (see Supplementary Materials Figure S1). The GO and KEGG analyses of the genes were performed to explore their biological function. Biological processes (BP) indicated that CXCR members are closely associated with immune function (Figure 2B); molecular function (MF) analysis indicated that CXCR members function in relation to T-cell receptor binding (Figure 2C). Cellular component (CC) indicated that CXCR members are closely associated with T-cell receptor complexes, as shown in the red box (Figure 2D). Meanwhile, in the KEGG pathway, we found that CXCRs-related genes were most significantly enriched in primary immunodeficiency (Figure 2E). These results suggested a correlation between CXCR members and immune infiltration in malignant GBM.

### 3.3. Association of CXCR Members with IDH1 Mutation Status and GBM Subtypes

**Figure 3:**
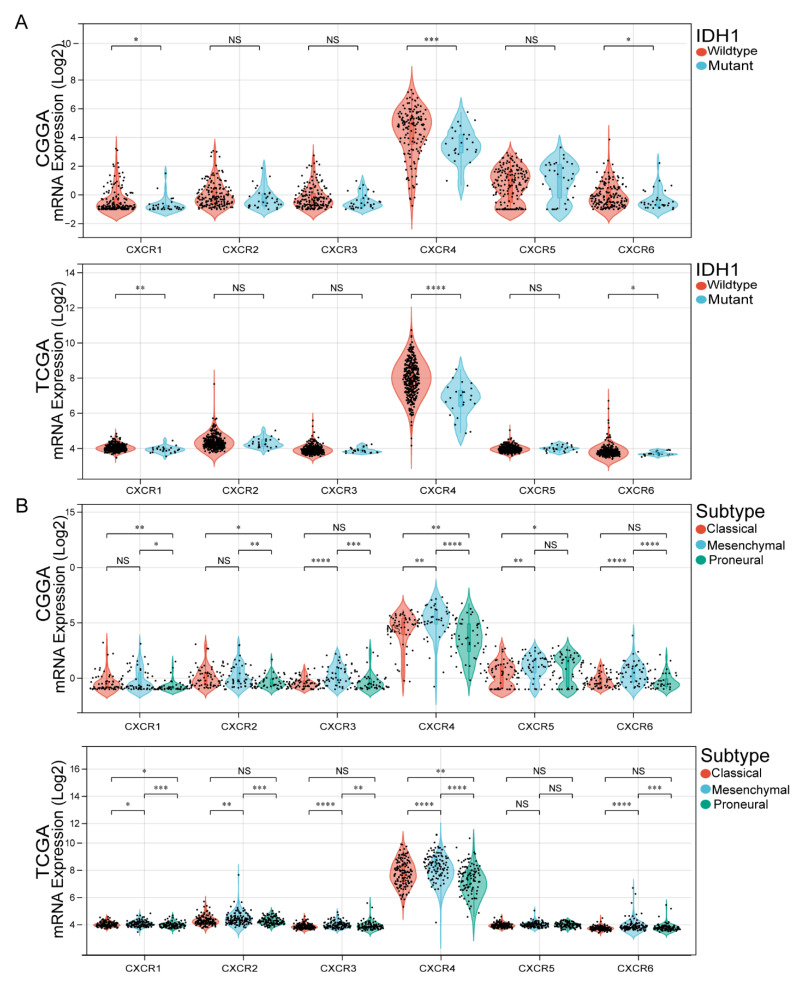
Correlation of CXCR members expression with IDH1 mutation status and GBM subtypes. (**A**), Expression of CXCR members (CXCR1/2/3/4/5/6) in classic, mesenchymal, and proneuronal GBM in TCGA and CGGA databases. (**B**), mRNA expression of CXCR members (CXCR1/2/3/4/5/6) in IDH1 wild-type and IDH1 mutant GBM in TCGA and CGGA databases. The black dots represent the expression of mRNA in the included population. * *p* < 0.05; ** *p* < 0.01; *** *p* < 0.001; **** *p* < 0.0001; ns not significant.

Isocitrate dehydrogenase (IDH) mutations have been identified as diagnostic and prognostic markers in GBM patients. To this end, we explored possible relationships between the CXCR members and the status of IDH mutations in the GBM from both the TCGA and CGGA databases. The results of analysis in TCGA database indicated that CXCR4 is significantly more expressed in IDH1 wild-type than in IDH1 mutant. Interestingly, the results of CXCR members analysis in CGGA database were similar to those in TCGA database (Figure 3A).

GBM is divided into three subtypes based on pathological features: proneuronal, classical and mesenchymal. Since the survival of GBM differs among subtypes, we further investigated CXCR members associated with GBM subtype. Therefore, we investigated whether the CXCR members are associated with GBM subtypes in the TCGA and CGGA databases. The obtained results showed (Figure 3B) that in TCGA, in CXCR1 and CXCR2, the expression of the classic and mesenchymal types was slightly higher than that of the proneuronal type; in CXCR3 and CXCR6, the expression of the mesenchymal type was significantly higher than that of the classical and proneuronal types. In CXCR4, the three subtypes were differentially expressed, with higher levels of CXCR4 mRNA expression in the mesenchymal. However, in CXCR5, the expression of mesenchymal and proneuronal was slightly higher than the expression of classical types. In CGGA, the mesenchymal expression of CXCR2, CXCR3, CXCR5, and CXCR6 were slightly higher than the expression of classical and proneuronal. In both CXCR1 and CXCR4, the three subtypes differed, but in CXCR4, the expression of mesenchymal was significantly higher than the expression of classical and proneuronal.

### 3.4. Relationship between Expression of CXCR Members and Prognosis of GBM Patients

**Figure 4:**
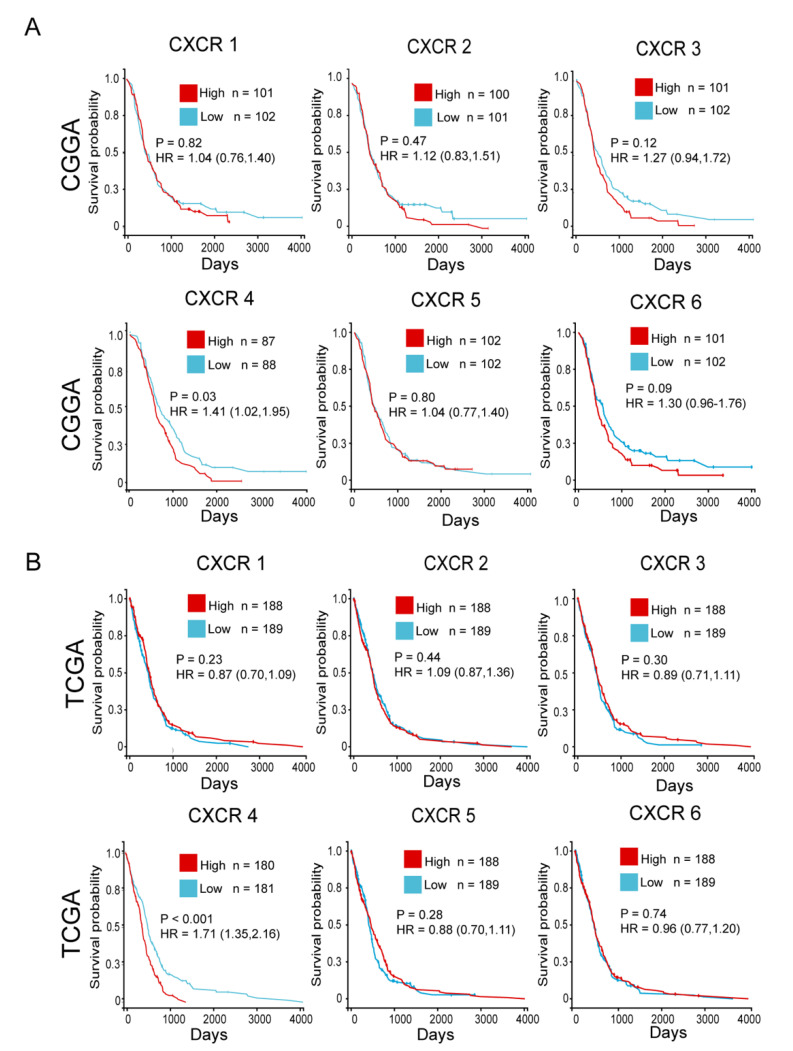
To investigate the prognostic value of the CXCR members in GBM. (**A**), Kaplan–Meier plots of overall survival of GBM patients with low and high CXCR members expression from the CGGA database. (**B**), Kaplan–Meier curves for overall survival of patients with CXCR members low and high expression GBM from the TCGA database, stratified by median. *p* < 0.05 indicates statistical significance.

To explore the prognostic value of CXCR members in GBM, we performed a comprehensive survival analysis of CXCR members in GBM patients included in the TCGA and CGGA databases. Our data showed (Figure 4A,B) that GBM patients with high CXCR4 expression had a shorter survival time, whereas CXCR1, CXCR2, CXCR3, CXCR5, and CXCR6 expression had no prognostic significance in GBM.

### 3.5. Construction of CXCR4-Based Nomogram and Risk Score Models for Predicting Survival of GBM Patients

**Figure 5:**
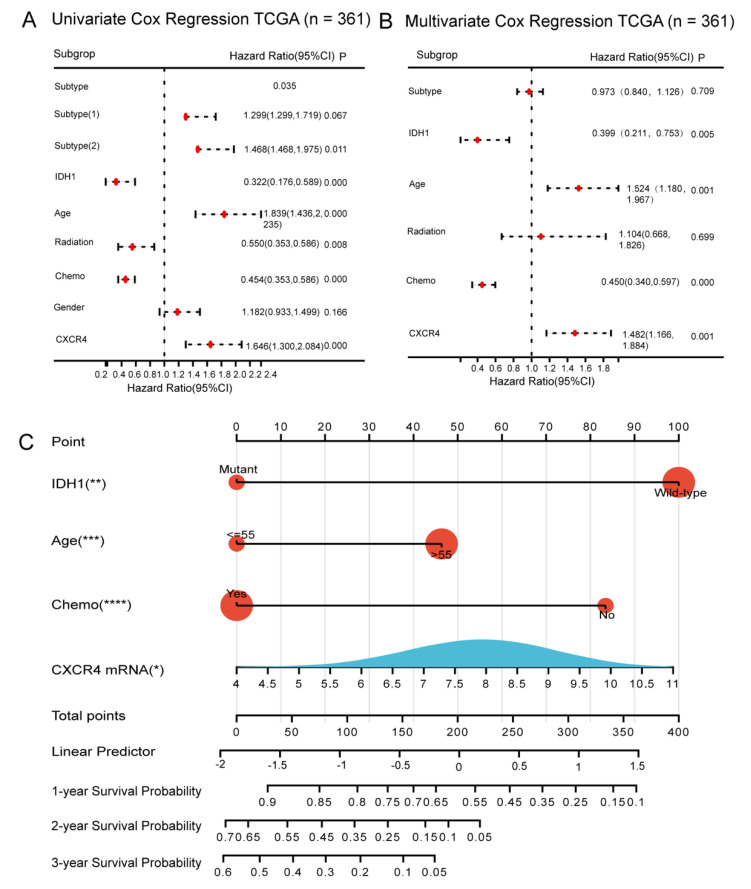
Construction of CXCR4-based nomogram and risk score models for predicting survival of GBM patients. (**A**,**B**), Forest plots showing univariate and multivariate Cox proportional risk ratios for age, sex, IDH1, Chemo, Radio, CXCR4 mRNA, and GBM subtypes in the TCGA database with their 95% confidence intervals. Red dots represent point estimates of HR values for each study. (**C**), Construction of nomogram from independent prognostic clinicopathological factors (CXCR4, IDH1 status, chemotherapy status, age) to predict 1-, 2-, and 3- year survival probabilities in GBM patients. * *p* < 0.05; ** *p* < 0.01; *** *p* < 0.001; **** *p* < 0.0001.

In this study, Cox survival regression analysis was used to determine the effect of CXCR4 on the prognosis of GBM patients, and we included age, sex, IDH1 mutation status, GBM subtype, chemotherapy status, radiotherapy status, and CXCR4 mRNA expression in the TCGA database for analysis. The results of univariate analysis showed that age, IDH1 mutation, CXCR4 mRNA, chemotherapy status, and radiotherapy status had a significant effect on survival in GBM patients (Figure 5A). A multivariate analysis incorporating these variables showed that GBM patients with high CXCR4 expression had a higher risk of poor survival compared to GBM patients with low CXCR4 expression (HR = 1.482, *p* < 0.001) (Figure 5B). It was confirmed that CXCR4 has an independent prognostic value in GBM patients. On this basis, we additionally developed a practical nomogram constructed from these independent clinicopathological variables to predict the survival of GBM patients at 1, 2, and 3 years (Figure 5C).

Next, we constructed a prognostic risk score model (PRSM) based on multifactorial Cox regression analysis. Using TCGA data as the training cohort, the risk score of each GBM patient was calculated by combining the coefficient-weighted scores of each variable. Afterwards, to validate the prognostic performance of the CXCR4-based risk score model in different populations, we included CGGA as an external validation cohort and calculated the risk score for each CGGA patient using the same formula.

### 3.6. Evaluation of CXCR4-Based Risk Scoring Models in Primary GBM

**Figure 6:**
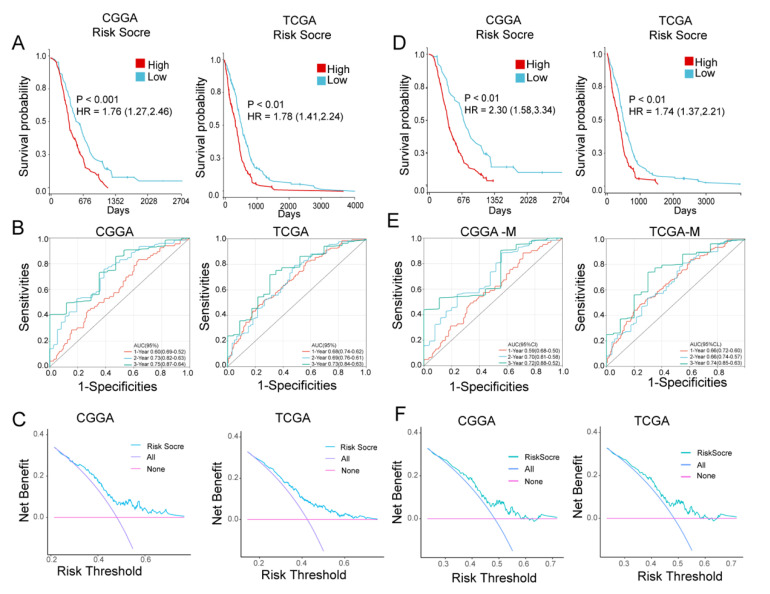
Evaluation of CXCR4-based risk score models in a primary GBM cohort. (**A**), Kaplan–Meier plots of overall survival of GBM patients with high- and low- risk scores from the TCGA and CGGA databases, stratified by median. (**B**), the time-dependent receiver operator characteristic curve (t-ROC) to assess the predictive accuracy of CXCR4-based prognostic risk score models for GBM patients in TCGA and CGGA databases. Predictive accuracy was calculated using 1-, 2-, and 3- year AUC. (**C**), decision curve analysis (DCA) was used to assess the net benefit of CXCR4-based prognostic risk scoring models in the TCGA and CGGA databases. (**D**), Kaplan–Meier plots of overall survival for patients with IDH1-WT GBM with high- and low- risk scores from the TCGA and CGGA databases, stratified by median. (**E**), accuracy of CXCR4-based prognostic risk score model in patients with IDH1-WT GBM in TCGA and CGGA databases. Predictive accuracy was calculated using 1-, 2-, and 3-year AUC. (**F**), DCA was used to assess the net benefit of CXCR4-based prognostic risk scoring models in patients with IDH1-WT GBM in the TCGA and CGGA databases.

To assess the accuracy of the CXCR4-based prognostic risk scoring model (PRSM) for survival prediction in patients with primary GBM, we validated it from different perspectives by Kaplan–Meier survival analysis, ROC, and DCA, respectively. First, we divided the TCGA population into high and low categories using the median as the cutoff value. In TCGA, Kaplan–Meier survival analysis showed that GBM patients with high-risk scores had shorter survival (HR = 1.78, *p* < 0.001). Similar findings were obtained in GBM patients with CGGA (Figure 6A), suggesting that the CXCR4-based risk score model is a useful method for the survival of GBM patients. Secondly, we calculated the area under the curve (AUC) by using time-dependent subject operating characteristic curve (t-ROC) analysis to assess the accuracy of the prognostic model. Our data show that the AUC of trained TCGA and tested CGGA PRSM is greater than 0.6 (Figure 6B), indicating that the accuracy of CXCR4 PRSM is higher. At the same time, the clinical efficacy of PRSM was evaluated by DCA. Our study found that CXCR4-based PRSM is clinically beneficial and significantly improves the prognosis of GBM patients (Figure 6C).

In the 2021 WHO classification, only GBM with IDH1 wild type (WT) is defined as GBM [21]. Therefore, we excluded glioblastomas with prior IDH1 mutations from the TCGA and CGGA glioblastoma datasets. Similar results were obtained by Kaplan–Meier analysis, t-ROC, and DCA assessment of IDH1-WT glioblastoma based on CXCR4 in PRSM. Our data showed that patients with IDH1-WT GBM with high-risk scores had shorter survival than patients with low-risk scores (Figure 6D–F). These results all show excellent accuracy and clinical benefit of the CXCR4-based prediction model.

### 3.7. CXCR4 Expression in the Infiltration Level of Immune Cells

**Figure 7:**
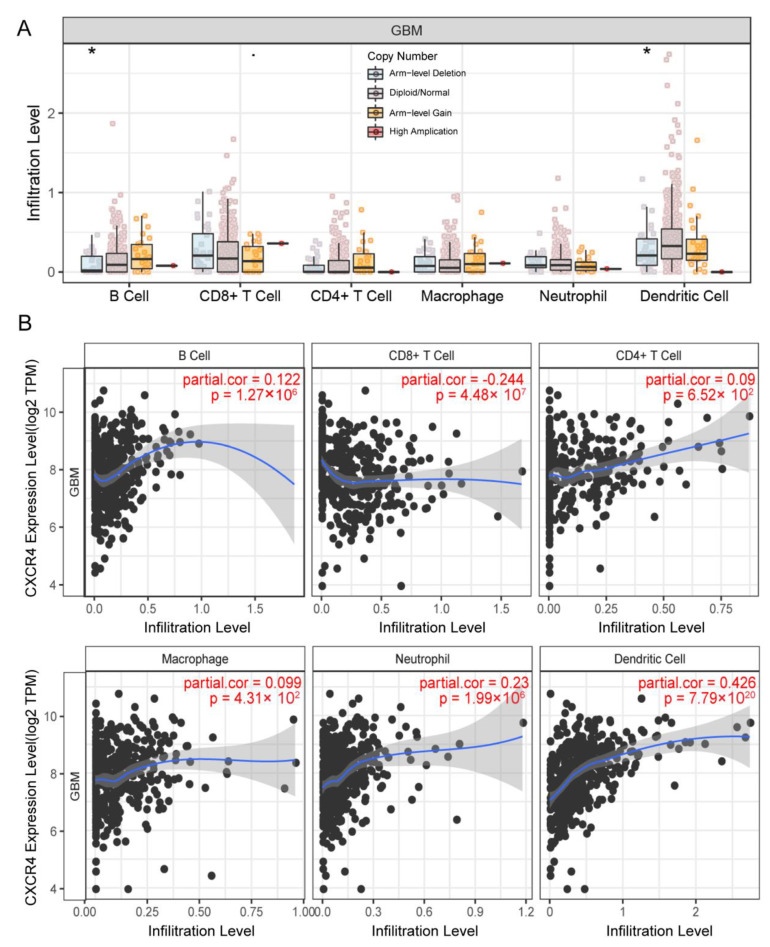
CXCR4-associated immune infiltration. (**A**), relationship between CXCR4 copy number alterations and infiltration levels of multiple immune cells (B cells, CD8+ T cells, CD4+ T cells, macrophages, neutrophils, dendritic cells). (**B**), relationship between CXCR4 mRNA expression and the level of infiltration of multiple immune cells (B cells, CD8+ T cells, CD4+ T cells, macrophages, neutrophils, dendritic cells). The blue line is a regression curve, representing the relationship between the horizontal and vertical coordinates, and the black dots represent the included population. * *p* < 0.05.

Due to the positive impact of immune cell therapy on clinical outcomes in GBM, we explored the correlation between CXCR4 and multiple immune cells by TIMER to further explore whether it has a positive impact on clinical prognosis in GBM [22]. It was found that arm-level gain alteration of CXCR4 was significantly correlated with immunity of B cells and dendritic cells (Figure 7A). At the mRNA level, CXCR4 expression positively correlated with B cells, CD4+ T cells, macrophages, neutrophils, and dendritic cells and negatively correlated with CD8+ T cells (Figure 7B), implying that CXCR4 has excellent potential for evaluating GBM immunotherapies [23,24].

### 3.8. Single Cell Analysis and Verification of Inflammatory Correlation

**Figure 8:**
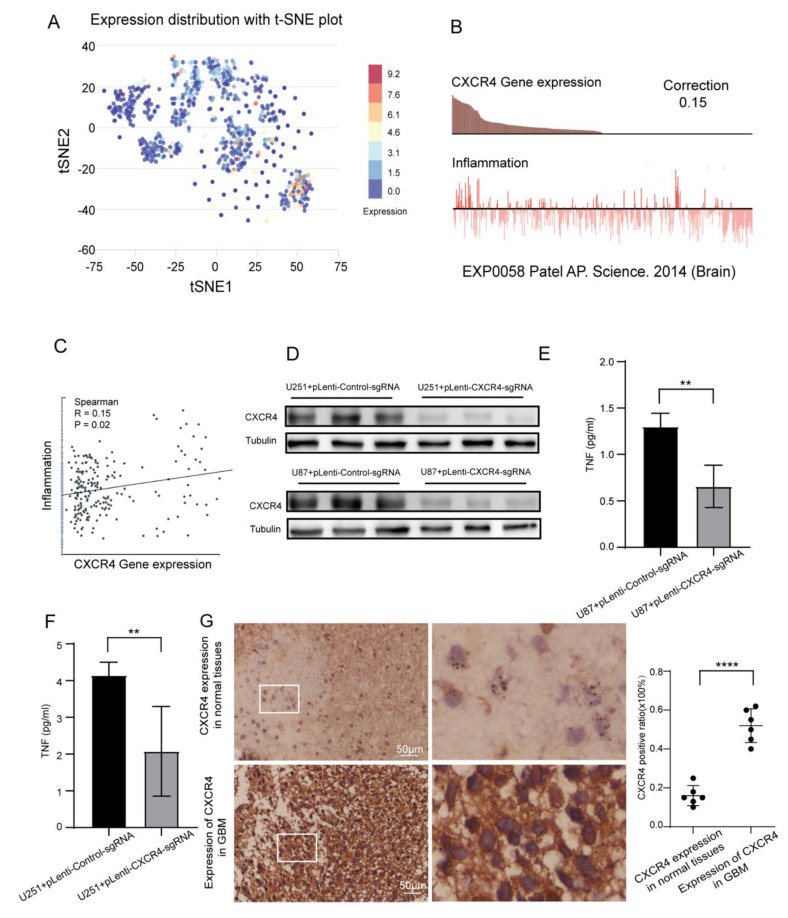
CXCR4 expression was closely associated with GBM tumor inflammation. (**A**), Single-cell analysis of CXCR4 differential expression distribution in GBM samples. (**B**,**C**), Expression range of CXCR4 in different GBM cells, correlation between CXCR4 and tumor inflammatory status, Spearman R = 0.15, *p* = 0.02. The red line represents the distribution of inflammatory phenotypes in the included population, and the black dots represent the included population. (**D**), Western blot detection of CXCR4 knockdown efficiency in U251 and U87 cells. (**E**,**F**), Inflammatory status of U251 and U87 cells with knockdown CXCR4 was assessed by Elisa. ** *p* < 0.01; **** *p* < 0.0001. (**G**), Immunostaining to detect CXCR4 expression in normal samples and GBM. Scale bar = 50 μm; data are expressed as mean ± standard deviation (mean ± SD).

We use CancerSEA (http://biocc.hrbmu.edu.cn/CancerSEA/home.jsp accessed on 15 March 2023) to explore the expression of CXCR4 on GBM function status analysis and visualization. The distribution of the CXCR4 expression is shown in Figure 8A. We further explored the relationship between CXCR4 expression and GBM phenotype. Moreover, we found that CXCR4 expression was positively correlated with tumor inflammation (Figure 8B,C). Therefore, we validated it with an in vitro experiment. We knocked down the expression of CXCR4 in glioma cell lines (U87 and U251) and used Western Blot to verify the knockdown effect (Figure 8D). Changes in the inflammatory factor TNF in knockdown cell lines were detected by Elisa. Our data showed that knockdown of CXCR4 significantly reduced the level of inflammation in glioma cells (Figure 8E,F), suggesting a positive correlation between CXCR4 and tumor cell inflammation in GBM. Finally, we used immunohistochemistry to detect whether CXCR4 was highly expressed in GBM and assessed the expression level of CXCR4 by a semi-quantitative assessment method, and the results were similarly confirmed (Figure 8G). Partial data are shown in Tables S3 and S4 of the Supplementary Materials.

## 4. Discussion

A growing number of studies have shown that the CXCR members are frequently involved in tumorigenesis and development and play an essential role in antitumor immunity [25,26]. However, the clinical value and application of the CXCR members in primary GBM has not been thoroughly clarified. Therefore, in this study, we comprehensively analyzed the mRNA expression changes of CXCR members using TCGA and CGGA databases to confirm their clinical prognostic value. The results showed that CXCR4 was highly expressed in GBM and had an independent prognostic value in glioblastoma. In addition, we constructed a CXCR4-based nomogram to predict survival in patients with primary GBM. Finally, we validated the role of CXCR4 in positively correlating with tumor inflammation.

Previous studies have suggested that CXCR1/2/3/4/7 may play a crucial role in gliomas, and have a significant impact on overall glioma survival [26]. It was confirmed that CXCR5 is not associated with glioma prognosis, which is consistent with the results of our present study. In addition, previous studies elaborated that CXCR6 expression in GBM and normal brain tissue is not significantly different [26]. This is consistent with our validation results in TCGA, but the CXCR6 expression is significantly different in CGGA. Possible reasons are, on the one hand, the heterogeneity of CXCR phenotypes in GBM patients [15], and on the other hand, the fact that we included data screening only WHO IV primary GBM samples, which may have contributed to this discrepancy. In our study, we screened primary glioblastoma data from the TCGA and CGGA databases and found that only CXCR3, CXCR4, and CXCR5 mRNA expression differed significantly between the two databases, suggesting that these members may be more closely linked in the onset and progression of GBM.

Based on the above findings, we explored the biological function of CXCR members by GO and KEGG and found a correlation with cellular immune infiltration, which provided a biological background for our subsequent studies. Later, we investigated the relationship of CXCR members with IDH mutation status and GBM subtypes to further explore the role of CXCRs in GBM. We further explored the prognostic value of CXCR members (CXCR1/2/3/4/5/6) in tumors by comprehensive survival analysis, from which CXCR4 was screened as an independent prognostic value, and explored significant differences in the level of immune cell infiltration and clinical features of CXCR4. 

In recent decades, CXCR4 has been increasingly studied in glioma [27]. CXCR4 is a cell surface chemokine receptor involved in many cell fate decisions such as growth, invasion, angiogenesis, and metastasis in a variety of malignancies [28]. The CXCL12/CXCR4 signaling pathway is activated in GBM and is critical for sustained glioma invasion, enhanced angiogenesis, and glioma stem cell migration [4]. CXCR4 antagonist (AMD3100) has been proposed as an anti-GBM therapeutic target that reduces the ability of glioma cell lines to survive and proliferate [6]. Furthermore, it has been suggested that CXCR4 is a strong predictor of poor prognosis in GBM patients and a clinical prognostic factor in glioma patients [29]. In the present study, we confirm this claim in more scientific and intuitive depth by means of bioinformatics techniques.

We found that CXCR4 is closely related to the prognosis of patients with GBM and preliminarily verified that CXCR4 is an important index to judge the prognosis of patients with GBM. Our previous study found that the prognosis of GBM with high expression of CXCR4 was worse, which was consistent with that reported in the literature [26]. Secondly, based on t-ROC and DCA to build a high-precision GBM prognosis prediction model to verify its application value in GBM patients. At the same time, based on IDH-WT (according to the 2021 WHO classification [21]), we screened it twice and got the same results as before.

Finally, through clinical specimens and in vitro studies of GBM patients, we found that CXCR4 was significantly overexpressed in GBM tissues and was closely associated with tumor inflammatory responses. We found that knockdown of CXCR4 in U87 and U251 had reduced levels of TNF. The possible reason for this is that knockdown of CXCR4 triggers different downstream signals in tumor cells, leading to a decrease in inflammatory factors. In addition, it has been shown that CXCR4 is consistently co-expressed with its ligand CXCL12 in glioblastoma multiforme [30] and that high levels of CXCL12 attract CXCR4-positive vascular and inflammatory cells and promote tumor cytokine secretion [23]. However, the specific mechanism needs further in-depth study.

Our previous work confirmed that CXCR4 is closely related to the prognosis of glioma patients, but there are still some deficiencies. First of all, because the project uses a retrospective experiment of two centers, there must be a preference for memory and choice in the process of the experiment. Secondly, the risk prognostic model would have better stability if it was in a larger dataset. Third, the inflammatory role of CXCR4 in GBM onset and development and its associated molecular mechanisms need further discussion.

## 5. Conclusions

CXCR members have a significant impact on immune infiltration and survival prognosis of glioblastoma, with CXCR4 as an independent prognostic factor positively correlated with inflammation in glioblastoma. This research will provide a new theoretical basis for the diagnosis, treatment, and prognosis of primary glioblastoma.

## Data Availability

The raw data of this study are available from the corresponding author upon reasonable request.

## Supplementary Materials

The following supporting information can be downloaded at: https://www.mdpi.com/article/10.3390/brainsci13081152/s1, Figure S1: The protein-protein interaction network (PPI); Table S1: Statistics reporting; Table S2: Full name of the cancer abbreviation in Figure 1; Table S3: Antibodies and Reagents; Table S4: Statistics reporting.

## Abbreviations

GBM, glioblastoma; CXCR, CXC chemokine receptor; CXCR4, C-X-C motif chemokine receptor 4; CNS, central nervous system; OS, overall survival; TCGA, The Cancer Genome Atlas; MDSC, myeloid-derived suppressor cells; PBS, phosphate buffer saline; ROC, receiver operating curves; IDH, isocitrate dehydrogenase; WT, wild-type; PRSM, prognostic risk scoring model; DCA, decision curve analysis; ELISA, enzyme-linked immunosorbent assay.
