# Supplementary material for: Development and Validation of CXCR4 Nomogram-Based Immune Infiltration/Tumor Inflammation in Primary Glioblastoma
Source: Brain Sci. 2023 Aug 1;13(8):1152. doi: 10.3390/brainsci13081152 (PMC10452349; doi:10.3390/brainsci13081152)
Supplement: Supplementary file 1 [file brainsci-13-01152-s001.zip › brainsci-2454685-supplementary.pdf]

Figure S1. The protein-protein interaction network (PPI).

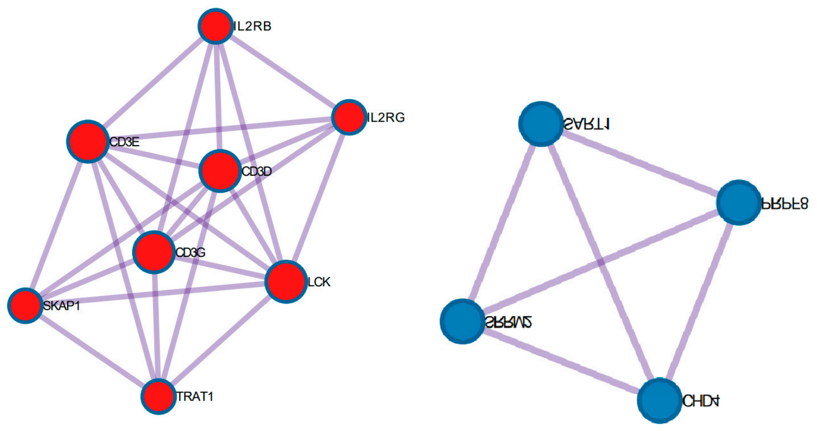

Color	MCODE	GO	Description	Log10(P)
	MCODE_1	M54	PID IL12 2PATHWAY	-14.8
	MCODE_1	hsa04658	Th1 and Th2 cell differentiation	-13.7
	MCODE_1	hsa04659	Th17 cell differentiation	-13.3
	MCODE_2	CORUM:1181	C complex spliceosome	-7.2
	MCODE_2	CORUM:351	Spliceosome	-6.4
	MCODE_2	R-HSA-72163	mRNA Splicing - Major Pathway	-5.9

Table S1. Statistics reporting.

Gene symbol	Gene ID	PCC
CXCR2	ENSG00000180871.7	0.98
S100A12	ENSG00000163221.8	0.96
FFAR2	ENSG00000126262.4	0.93
RP4-568C11.4	ENSG00000274173.1	0.91
RN7SL653P	ENSG00000239794.3	0.9
U3	ENSG00000281710.1	0.9
RP11-666A8.11	ENSG00000267084.1	0.9
LINC01360	ENSG00000233973.5	0.9
SNX19P1	ENSG00000228184.1	0.9
RPS4XP12	ENSG00000244400.2	0.9
OR4A7P	ENSG00000255349.1	0.9
RP4-591B8.3	ENSG00000271419.1	0.9
PRSS3P3	ENSG00000277083.1	0.9
RP11-325O24.6	ENSG00000281248.1	0.9
CT83	ENSG00000204019.4	0.9
OR2T3	ENSG00000196539.3	0.9
PRPF19P1	ENSG00000264685.1	0.9
RP11-353N14.3	ENSG00000262343.1	0.9

AC093698.4	ENSG00000224529.1	0.9
AC055736.3	ENSG00000281194.1	0.9
CXCR1	ENSG00000163464.7	0.98
S100A12	ENSG00000163221.8	0.93
RP4-568C11.4	ENSG00000274173.1	0.93
FFAR2	ENSG00000126262.4	0.93
PRSS3P3	ENSG00000277083.1	0.92
RP11-325O24.6	ENSG00000281248.1	0.92
RN7SL653P	ENSG00000239794.3	0.92
RP11-666A8.11	ENSG00000267084.1	0.92
U3	ENSG00000281710.1	0.92
RP11-305F5.2	ENSG00000243016.1	0.92
CT83	ENSG00000204019.4	0.92
LINC01360	ENSG00000233973.5	0.92
AC093698.4	ENSG00000224529.1	0.92
AC055736.3	ENSG00000281194.1	0.92
SNX19P1	ENSG00000228184.1	0.92
OR8J1	ENSG00000172487.3	0.92
RP11-799D4.2	ENSG00000266981.1	0.92
RPS4XP12	ENSG00000244400.2	0.92
OR4A7P	ENSG00000255349.1	0.92
RP4-591B8.3	ENSG00000271419.1	0.92
CD3E	ENSG00000198851.9	0.94
IKZF3	ENSG00000161405.16	0.94
LCK	ENSG00000182866.16	0.93
CD2	ENSG00000116824.4	0.93
SIRPG	ENSG00000089012.14	0.92
TRBV25-1	ENSG00000211751.7	0.92
CD27	ENSG00000139193.3	0.91
ICOS	ENSG00000163600.12	0.91
PTPRCAP	ENSG00000213402.2	0.89
IL2RG	ENSG00000147168.12	0.89
PYHIN1	ENSG00000163564.14	0.89
IL2RB	ENSG00000100385.13	0.88
SLA2	ENSG00000101082.13	0.88
P2RY10	ENSG00000078589.12	0.88
GZMK	ENSG00000113088.5	0.87
SIT1	ENSG00000137078.8	0.87
CD6	ENSG00000013725.14	0.87
SH2D1A	ENSG00000183918.14	0.86
SLAMF7	ENSG00000026751.16	0.86
TRAT1	ENSG00000163519.13	0.85
FGF5	ENSG00000138675.16	0.6

PIK3C3	ENSG00000078142.11	0.58
RP1-244F24.1	ENSG00000271857.1	0.57
RP11-264B14.1	ENSG00000267207.1	0.57
GPR39	ENSG00000183840.6	0.57
RP11-205M5.3	ENSG00000280064.1	0.57
KLF5	ENSG00000102554.13	0.56
NKX2-6	ENSG00000180053.7	0.56
RP11-59D5__B.2	ENSG00000236345.1	0.56
HGF	ENSG00000019991.15	0.55
C9orf92	ENSG00000205549.8	0.54
CYP2S1	ENSG00000167600.13	0.54
RNF144B	ENSG00000137393.9	0.54
NDFIP1P1	ENSG00000255892.1	0.53
SULT1C2	ENSG00000198203.9	0.53
RN7SKP154	ENSG00000222068.1	0.53
RP11-384F7.1	ENSG00000243276.5	0.53
RP11-316I3.1	ENSG00000237283.1	0.53
MKX-AS1	ENSG00000230500.1	0.53
H2BFS	ENSG00000234289.5	0.53
CEP131	ENSG00000141577.13	0.69
PRRC2A	ENSG00000204469.12	0.68
SRRM2	ENSG00000167978.16	0.66
INTS1	ENSG00000164880.15	0.66
SART1	ENSG00000175467.14	0.66
GAK	ENSG00000178950.16	0.66
USP20	ENSG00000136878.12	0.65
HGS	ENSG00000185359.12	0.65
ZBTB17	ENSG00000116809.11	0.65
ATXN2L	ENSG00000168488.18	0.65
CHD4	ENSG00000111642.14	0.65
TRAPPC12	ENSG00000171853.15	0.64
PRPF8	ENSG00000174231.16	0.64
RP11-2C24.3	ENSG00000280211.1	0.64
RP11-488C13.5	ENSG00000258301.3	0.64
GTF3C1	ENSG00000077235.17	0.64
MED24	ENSG00000008838.17	0.64
IGHMBP2	ENSG00000132740.8	0.63
SPTAN1	ENSG00000197694.13	0.63
DROSHA	ENSG00000113360.16	0.63
CD3D	ENSG00000167286.9	0.93
CD2	ENSG00000116824.4	0.9
PTPRCAP	ENSG00000213402.2	0.87
LCK	ENSG00000182866.16	0.86

TRBV25-1	ENSG00000211751.7	0.86
SIT1	ENSG00000137078.8	0.85
CD3E	ENSG00000198851.9	0.85
CD27	ENSG00000139193.3	0.84
GZMA	ENSG00000145649.7	0.83
GZMH	ENSG00000100450.12	0.83
CCL5	ENSG00000271503.5	0.83
ICOS	ENSG00000163600.12	0.82
SKAP1	ENSG00000141293.15	0.81
TRBV19	ENSG00000211746.3	0.81
CD96	ENSG00000153283.12	0.8
CD3G	ENSG00000160654.9	0.8
SIRPG	ENSG00000089012.14	0.79
SLA2	ENSG00000101082.13	0.79
PYHIN1	ENSG00000163564.14	0.79
KLRB1	ENSG00000111796.3	0.78

Table S2. Full name of the cancer abbreviation in Figure 1.

Cohort	Name of disease	Cohort	Name of disease
TCGA-ACC	Adrenocortical carcinoma	TCGA-LUAD	Lung adenocarcinoma
TCGA-BLCA	Bladder Urothelial Carcinoma	TCGA-LUSC	Lung squamous cell carcinoma
TCGA-BRCA	Breast invasive carcinoma	TCGA-MESO	Mesothelioma
TCGA-CESC	Cervical squamous cell carcinoma and endocervical adenocarcinoma	TCGA-OV	Ovarian serous cystadenocarcinoma
TCGA-CHOL	Cholangiocarcinoma	TCGA-PAAD	Pancreatic adenocarcinoma
TCGA-COAD	Colon adenocarcinoma	TCGA-PCPG	Pheochromocytoma and Paraganglioma
TCGA-COADREAD	Colon adenocarcinoma/Rectum adenocarcinoma Esophageal carcinoma	TCGA-PRAD	Prostate adenocarcinoma
TCGA-DLBC	Lymphoid Neoplasm Diffuse Large B-cell Lymphoma	TCGA-READ	Rectum adenocarcinoma
TCGA-ESCA	Esophageal carcinoma	TCGA-SARC	Sarcoma
TCGA-FPPP	FFPE Pilot Phase II	TCGA-STAD	Stomach adenocarcinoma
TCGA-GBM	Glioblastoma multiforme	TCGA-SKCM	Skin Cutaneous

			Melanoma
TCGA-GBMLGG	Glioma	TCGA-STES	Stomach and Esophageal carcinoma
TCGA-HNSC	Head and Neck squamous cell carcinoma	TCGA-TGCT	Testicular Germ Cell Tumors
TCGA-KICH	Kidney Chromophobe	TCGA-THCA	Thyroid carcinoma
TCGA-KIPAN	Pan-kidney cohort (KICH+KIRC+KIRP)	TCGA-THYM	Thymoma
TCGA-KIRC	Kidney renal clear cell carcinoma	TCGA-UCEC	Uterine Corpus Endometrial Carcinoma
TCGA-KIRP	Kidney renal papillary cell carcinoma	TCGA-UCS	Uterine Carcinosarcoma
TCGA-LAML	Acute Myeloid Leukemia	TCGA-UVM	Uveal Melanoma
TCGA-LGG	Brain Lower Grade Glioma	TARGET-OS	Osteosarcoma
TCGA-LIHC	Liver hepatocellular carcinoma	TARGET-ALL	Acute Lymphoblastic Leukemia
TARGET-NB	Neuroblastoma	TARGET-WT	High-Risk Wilms Tumor

Table S3. Antibodies and Reagents.

Antibody/ Reagents	Full name	Specificity	Citation	Type	Host
CXCR4	Anti-CXCR4 Antibody	Mouse, Rat, Human	AF6621	monoclonal antibody	Rabbit
β-tubulin	Tubulin beta Antibody	Mouse, Rat, Human	AF7011	monoclonal antibody	Mouse
Human TNF-α ELISA Kit	Human TNF-α (Tumor Necrosis Factor Alpha) ELISA Kit	Human	EH0302		Human
Immunostaining reagents	GTVision TM III Detection System/ Mo&Rb(Including DAB)	Mouse, Rat	GK500705		Mouse, Rat
pLenti-CXCR4-sgRNA			L23050		

pLenti-Control-sgRNA	L00011
U87MG	YS190C1
U251	ZQ0053

Table S4. Statistics reporting.

Figure	n	NAME	DATA STRUCTURE	TEST USED	STATISTIC	P VALUE
**8D**	6	CXCR4 positive ratio	Normal distribution	Unpaired t-test	F=2.803	P<0.0001
**8F**	6	U87-TNF (pg/ml)	Normal distribution	Unpaired t-test	F =2.515	P=0.0030
**8F**	6	U251-TNF(pg/ml）	Normal distribution	Unpaired t-test	F =9.826	P=0.0014
